# Seminoma in a 46, XY patient with 17α‐hydroxylase deficiency

**DOI:** 10.1002/iju5.12737

**Published:** 2024-05-20

**Authors:** Ken Maekawa, Yousuke Shimizu, Koken Hayashi, Shotaro Hatano, Yasuyuki Miyauchi, Takaki Sakurai, Kenji Mitsumori, Hiroyuki Onishi

**Affiliations:** ^1^ Department of Urology Osaka Red Cross Hospital Osaka Japan; ^2^ Department of Pathology Osaka Red Cross Hospital Osaka Japan

**Keywords:** 17α‐hydroxylase deficiency, disorder of sex development, prophylactic gonadectomy, seminoma, XY karyotype

## Abstract

**Introduction:**

17α‐Hydroxylase deficiency is a very rare disease reported to be associated with a risk of gonadal malignancy. We herein report a rare case of seminoma in a 46, XY patient with 17α‐hydroxylase deficiency.

**Case presentation:**

A 52‐year‐old woman presented with a 9‐cm pelvic tumor. At age 14, she had been identified as having the XY karyotype and 17α‐hydroxylase deficiency. However, she was not informed and did not consult the urology department. Laparoscopic gonadectomy was performed at the latest consultation, and seminoma was diagnosed.

**Conclusion:**

This is the third reported case of testicular tumor and the first of germ cell tumor in a 46, XY patient with 17α‐hydroxylase deficiency. Given the rarity and the risk of gonadal malignancy associated with 17α‐hydroxylase deficiency, the involvement of multidisciplinary specialists and prophylactic gonadectomy is considered crucial in its management.

**Keynote message**

We experienced a rare case of seminoma in a 46, XY patient with 17α‐hydroxylase deficiency. Because of the rarity of 46, XY patients with 17α‐hydroxylase deficiency and the associated risk of gonadal malignancy, a multidisciplinary approach and prophylactic gonadectomy are considered necessary.

**Abbreviations & Acronyms**
17OHD: 17 alpha hydroxylase deficiency
17‐OHP: 17 alpha hydroxyprogesterone
ACTH: adrenocorticotrophic hormone
AFP: alpha‐fetoprotein
CAH: congenital adrenal hyperplasia
CAIS: complete androgen insensitivity syndrome
CT: computed tomography
DHEA: dehydroepiandrosterone
DOC: 11‐deoxycorticosterone
FSH: follicle‐stimulating hormone
GD: pure gonadal dysgenesis
hCG: human chorionic gonadotropin
LDH: lactate dehydrogenase
LH: luteinizing hormone
MRI: magnetic resonance imaging
PAIS: partial androgen insensitivity syndrome

## Introduction

17OHD is a very rare disease, representing only 1% of CAH.^1^ Most patients with a 46, XY karyotype and 17OHD (46, XY 17OHD) are phenotypically female, and their social gender is typically female.^2^ The testes of 46, XY 17OHD patients are located in the abdomen or inguinal canal and have a risk of malignancy.^2^, ^3^ Two reports of malignant testicular tumors (Leydig and Sertoli cell tumors) in 46, XY 17OHD patients have been published.^2^ We report the third such case, here involving a seminoma. To our knowledge, this is the first case of germ cell tumor in a 46, XY 17OHD patient.

## Case presentation

**Fig. 1:**
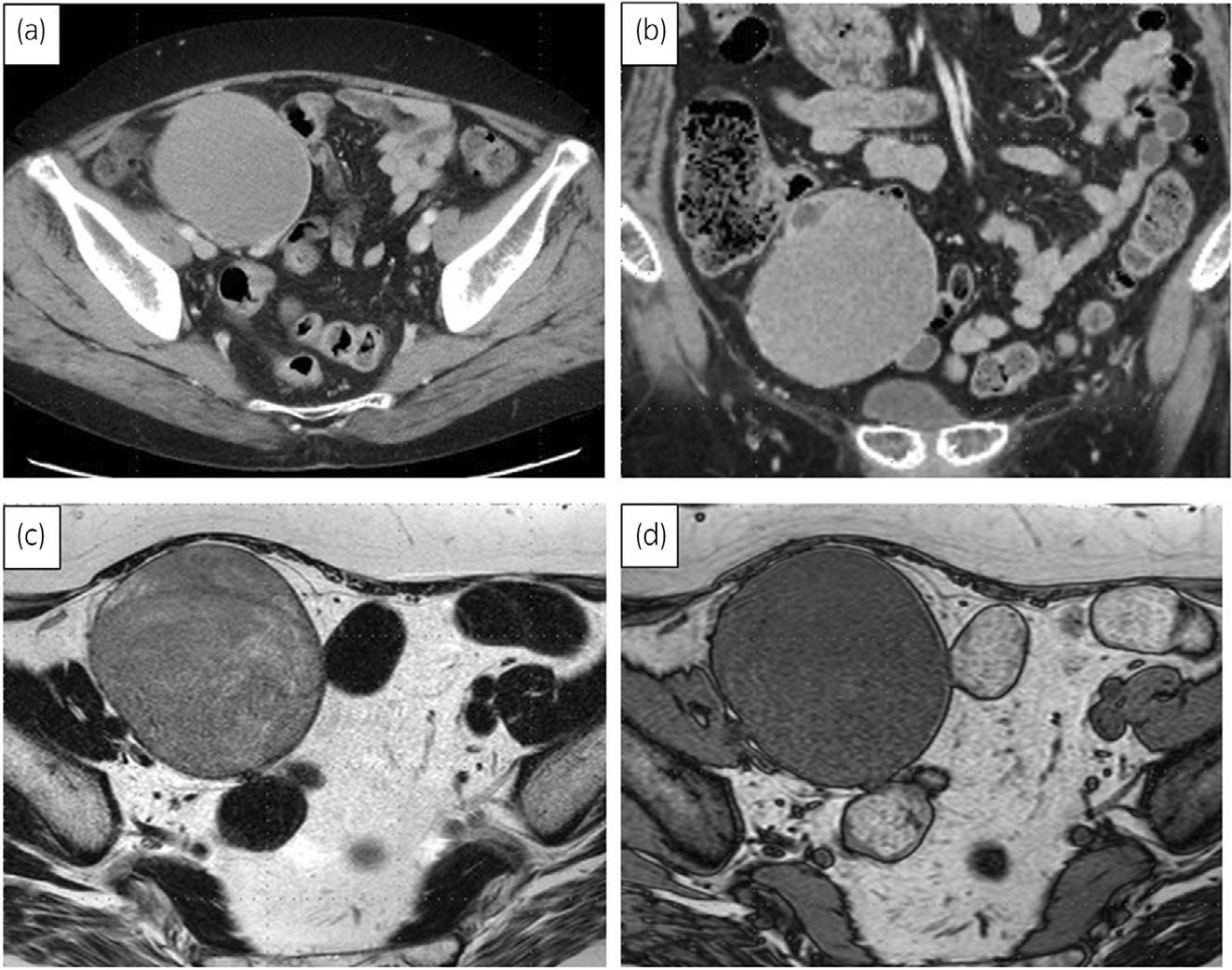
(a, b) CT revealed a 90‐mm tumor in the right pelvic region. (a) A horizontal section, and (b) a coronal section. (c, d) MRI revealed a 90‐mm solid tumor in the right pelvic region that was continuous with the right testicular artery and vein, and exhibited low signal intensity on T1‐weighted images and relatively homogeneous low signal intensity on T2‐weighted images. (c) A T1‐weighted image and (d) a T2‐weighted image.

**Fig. 2:**
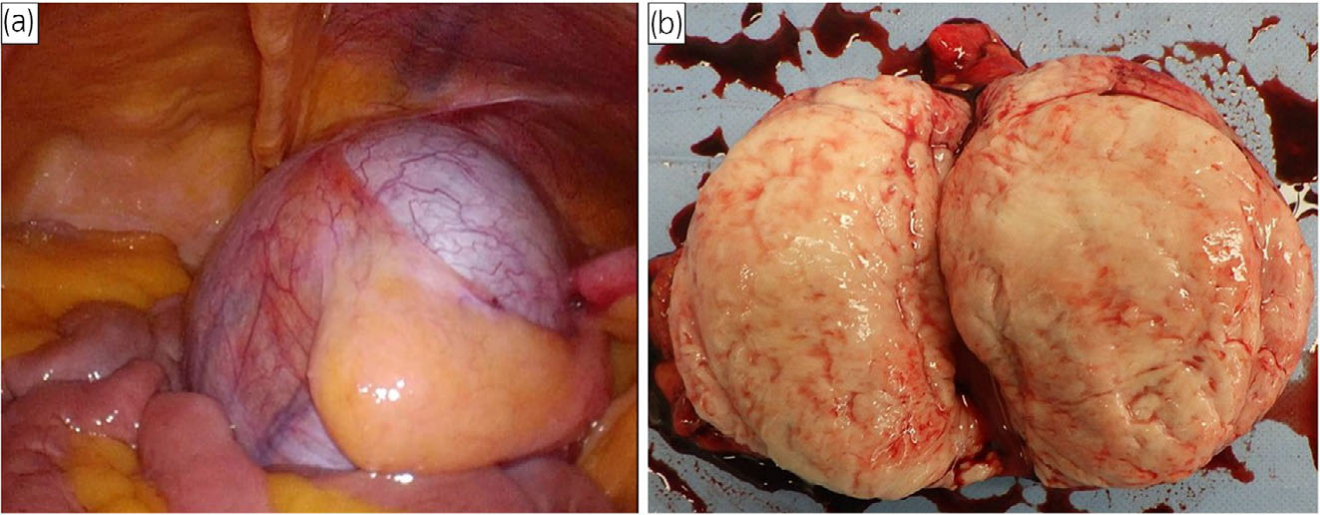
(a) Laparoscopic findings of the right testicular tumor. (b) Macroscopic cut surface of the right testicular tumor.

**Fig. 3:**
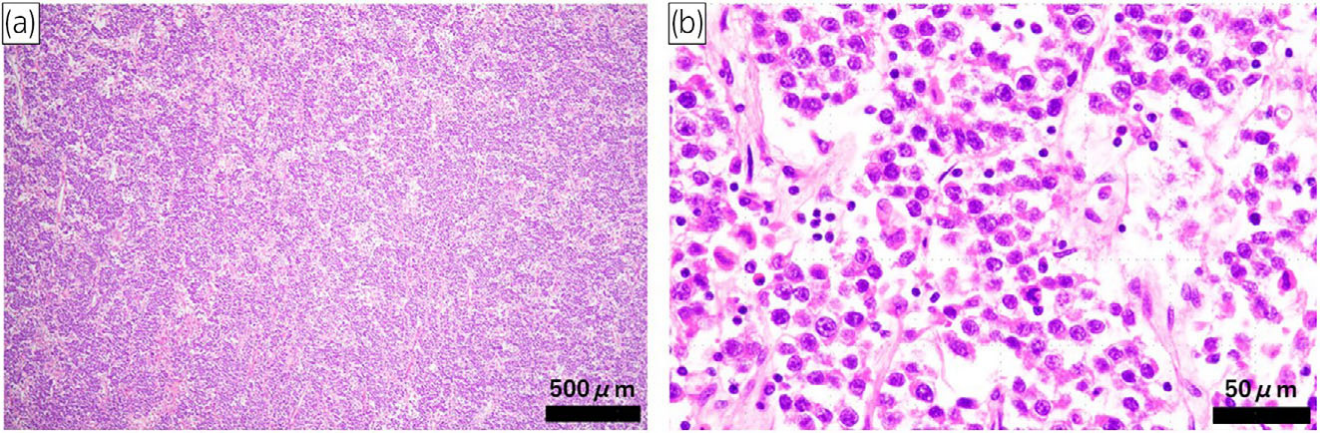
Pathological findings of the right testicular tumor. The right testicular tumor reveals typical pure form of seminoma. The tumor exhibited a two‐cell pattern with large round nuclei, prominent mitotic figures, and infiltration of mature lymphocytes (HE stain; a: ×4, b: ×40).

A 52‐year‐old woman was referred to our hospital with lower abdominal discomfort. CT revealed a 9‐cm tumor in the right pelvic region (Fig. 1a,b). At age 10, she had been diagnosed with hypertension. Laboratory findings showed low plasma renin activity (<0.3 ng/mL/h) and elevated serum aldosterone (498.7 pg/mL), suggesting primary aldosteronism. Owing to persistent hypertension until 14 years old and the presence of amenorrhea, she was admitted for further examination. Blood tests revealed elevated levels of ACTH of 174 pg/mL (normal 7.2–63.3 pg/mL), FSH 177.8 mIU/mL (2.0–8.3 mIU/mL), LH 274 mIU/mL (0.52–7.8 mIU/mL), pregnenolone 3.0 μg/mL (0.1–1.0 ng/mL), progesterone 6.1 ng/mL (0.2–1.4 ng/mL), and DOC 2.94 ng/mL (0.08–0.28 ng/mL). Low values were observed for cortisol of 1.2 μg/dL (5–22 μg/dL), 17‐OHP 0.24 ng/mL (0.05–1.60 ng/mL), DHEA 0.8 ng/mL (55–342 μg/dL), and testosterone 0.07 ng/mL (1.31–8.71 ng/mL), leading to a diagnosis of 17OHD. Chromosomal analysis confirmed an XY karyotype. Treatment with dexamethasone normalized blood pressure by suppressing ACTH and aldosterone without antihypertensive medication, and it is currently being continued. At the time of this admission, the patient was 178.1 cm tall and weighed 81.5 kg. External genitalia were typically female, with a short blind‐ending vagina. The patient's social gender is female. General blood tests showed no abnormalities. The hormone and tumor marker levels were as follows: FSH 92.5 mIU/mL (2.0–8.3 mIU/mL), LH 20.5 mIU/mL (0.52–7.8 mIU/mL), hCG 46.7 IU/L (<2.0 IU/L), AFP 2.5 ng/mL (<10 ng/mL), and LDH 234 U/L (119–229 U/L). MRI revealed a 90‐mm tumor. Low signal intensity was exhibited on T1‐weighted images, and relatively homogeneous low signal intensity was found on T2‐weighted images (Fig. 1c,d), leading to a diagnosis of malignant transformation of right undescended testis. CT showed no metastasis, and a 14‐mm left testis was found in the left inguinal canal. A diagnosis of cT1N0M0 testicular tumor was made. A laparoscopic bilateral gonadectomy was performed via a transperitoneal approach. The tumor was removed after resecting the right testicular vessels (Fig. 2a). The left testis in the inguinal canal was removed, given the risk of malignancy. In total, the operation took 224 min, with minimal blood loss. Macroscopically, the tumor was 90 mm, well‐defined, nodular, and white (Fig. 2b). Histologically, it exhibited a two‐cell pattern with large round nuclei, prominent mitotic figures, and mature lymphocyte infiltration, being diagnosed as seminoma (Fig. 3). Infiltration into surrounding adipose tissue was observed, and the resection margins were negative. Pathological stage was pT2. In the left testis, there was Leydig cell hyperplasia, and no malignant findings were observed. The patient was discharged 8 days after surgery without complications. Adjuvant chemotherapy was not administered. After the surgery, the tumor markers have normalized, and there have been no signs of recurrence for 8 months.

## Discussion

17OHD is a rare form of CAH with an incidence of approximately 1 in 50,000.^1^ It is an autosomal recessive disorder caused by CYP17A1 gene mutations.^4^ 17OHD affects the enzyme's activity, leading to disordered corticosteroid, estrogen, and testosterone synthesis.^5^ Decreased cortisol secretion increases the production of ACTH, which stimulates the synthesis of DOC and corticosterone, causing hypertension and hypokalemia.^6^ Corticosterone has weak glucocorticoid activity, and adrenal insufficiency symptoms are rare.^7^ In 46, XY 17OHD patients, anti‐Müllerian hormone is normally secreted in the embryonic stage, causing regression of the fallopian tubes, uterus, and upper third of the vagina.^8^ The internal genitalia are hypoplastic testes, and the external genitalia are female or ambiguous owing to the absence of androgen.^9^ The social gender is almost always female.^10^ Diagnosis is typically made by puberty, with cases presenting hypertension, hypokalemia, abnormal external genitalia, and amenorrhea.^11^ Disorders causing XY females include 46, XY GD (pure XYGD), 45,X/46,XY mixed gonadal dysgenesis (mixed GD), CAIS, PAIS, and 17OHD.^3^ Jiang *et al*. reported 202 cases of prophylactic gonadectomy performed on XY females, with a mean age of 20.6 years.^3^ Their testicular tumors were classified as follows: 22.4% pure XYGD (15/67), 10.9% mixed GD (5/46), 27.1% CAIS (13/48), 9.5% PAIS (2/21), and 10% 17OHD (2/20).^3^ The pathological findings of the two 46, XY 17OHD cases were Leydig and Sertoli cell tumors, at 16 and 17 years old, respectively, which are classified as sex cord‐stromal tumors.^2^ Gonads of XY females of any type are at risk of malignancy, highlighting the need for timely gonadectomy.^3^ Michael J Mathers *et al*. reported that the carcinogenicity of intra‐abdominal gonads is five times higher than that of inguinal cryptorchidism.^12^ We experienced a seminoma in a 46, XY 17OHD patient, which is the first report of a germ cell tumor in such a case. Owing to 17OHD's rarity, there are no clear treatment guidelines for gonadectomy.^3^ However, Jiang *et al*. concluded that gonadectomy should be performed at the time of diagnosis in adulthood.^2^ There were two difficulties in the current case. First, despite having been diagnosed with 17OHD at age 14, no consultation for gonadal treatment at the urology department was performed. Second, only the parents were notified of the results of the chromosome test. The patient learned of the results for the first time during the currently reported consultation at the age of 52. Owing to the highly sensitive nature of issues related to gender identity, it is important for mental health support to be provided by professionals such as genetic counselors in such cases. Because 17OHD is a rare disease, a multidisciplinary approach involving endocrinologists, urologists, gynecologists, and genetic counselors is considered essential.^9^

## Conclusion

We experienced a seminoma in a patient with 46, XY 17OHD. This is the third reported case of a testicular tumor and the first of a germ cell tumor in a 46, XY 17OHD patient. Because of the risk of malignancy in such patients, prophylactic gonadectomy is considered necessary. Owing to the rarity of 46, XY 17OHD, the involvement of multidisciplinary specialists is deemed crucial in its management.

## Author contributions

Ken Maekawa: Writing – original draft. Yousuke Shimizu: Supervision. Koken Hayashi: Software. Shotaro Hatano: Resources. Yasuyuki Miyauchi: Methodology. Takaki Sakurai: Data curation. Kenji Mitsumori: Methodology. Hiroyuki Onishi: Funding acquisition.

## Conflict of interest

The authors declare no conflict of interest.

## Approval of the research protocol by an Institutional Reviewer Board

Not applicable.

## Informed consent

We obtained written informed consent from the patient.

## Registry and the Registration No. of the study/trial

Not applicable.
